# Inhibition of glucose metabolism prevents glycosylation of the glutamine transporter ASCT2 and promotes compensatory LAT1 upregulation in leukemia cells

**DOI:** 10.18632/oncotarget.10131

**Published:** 2016-06-17

**Authors:** Florence Polet, Ruben Martherus, Cyril Corbet, Adan Pinto, Olivier Feron

**Affiliations:** ^1^ Pole of Pharmacology and Therapeutics, Institut de Recherche Expérimentale et Clinique (IREC), Université Catholique de Louvain, B-1200 Brussels, Belgium

**Keywords:** leukemia, ASCT2, LAT1, glycosylation, metabolism

## Abstract

Leukemia cells are highly dependent on glucose and glutamine as bioenergetic and biosynthetic fuels. Inhibition of the metabolism of glucose but also of glutamine is thus proposed as a therapeutic modality to block leukemia cell growth. Since glucose also supports protein glycosylation, we wondered whether part of the growth inhibitory effects resulting from glycolysis inhibition could indirectly result from a defect in glycosylation of glutamine transporters. We found that ASCT2/SLC1A5, a major glutamine transporter, was indeed deglycosylated upon glucose deprivation and 2-deoxyglucose exposure in HL-60 and K-562 leukemia cells. Inhibition of glycosylation by these modalities as well as by the *bona fide* glycosylation inhibitor tunicamycin however marginally influenced glutamine transport and did not impact on ASCT2 subcellular location. This work eventually unraveled the dispensability of ASCT2 to support HL-60 and K-562 leukemia cell growth and identified the upregulation of the neutral amino acid antiporter LAT1/SLC7A5 as a mechanism counteracting the inhibition of glycosylation. Pharmacological inhibition of LAT1 increased the growth inhibitory effects and the inactivation of the mTOR pathway resulting from glycosylation defects, an effect further emphasized during the regrowth period post-treatment with tunicamycin. In conclusion, this study points towards the underestimated impact of glycosylation inhibition in the interpretation of metabolic alterations resulting from glycolysis inhibition, and identifies LAT1 as a therapeutic target to prevent compensatory mechanisms induced by alterations in the glycosylating process.

## INTRODUCTION

Many transporters and receptors are N-glycosylated, and their cell-surface expression depends on proper folding and the degree of N-glycan branching [1]. Glucose is necessary for glycosylation through its utilization in the hexosamine biosynthetic pathway. The hexosamine pathway branches off from glycolysis at fructose-6-phosphate and produces UDP-N-acetylglucosamine (UDP-GlcNAc), the substrate for N-glycosylation [2]. Interestingly, while inhibitors of glycolysis are proposed to inhibit tumor cell growth through the blockade of a preferential source of energy fuels in cancer cells and tumor stroma cells [3–6], little is known about the contribution of the inhibition of glycosylation on tumor metabolism.

Other bioenergetic and biosynthetic fuels than glucose are taken up into cells by a variety of transporters that are potentially glycosylated. In leukemia, we and others have identified glutamine as a critical nutrient for cell growth [7–10]. Indeed, while leukemia cells have been described for years as the prototypical glycolytic cells, we know today that leukemia cells are highly dependent on glutamine to feed the TCA cycle. Although glutamine is a non-essential amino acid in normal cells, the demand for glutamine is dramatically increased throughout malignant transformation to support increased metabolic demands, in particular the need of anabolic substrates for macromolecule biosynthesis [11, 12]. Numerous amino acid transporters have been reported to transport glutamine. Glutamine transporters belong to different protein families nowadays classified according to the SLC nomenclature, the most frequent belonging to the SLC1, 6, 7, and 38 families [13, 14]. Most transporters share specificity for other neutral or cationic amino acids. Na^+^-dependent symporters capture glutamine while antiporters regulate the pools of glutamine and other amino acids. SLC1A5 (also known as ASCT2) is the most described glutamine transporter in cancer cells. Blocking ASCT2 to prevent glutamine uptake has been shown to successfully prevent the growth of melanoma [15], prostate cancer [16], non-small cell lung cancer [17], triple-negative basal-like breast cancer [18] and acute myeloid leukemia [8]. Part of the explanation for the prominent role of ASCT2 in cancer comes from the coupling of this transporter with a neutral amino acids antiporter [19]. Indeed, according to a two-step process, part of the glutamine taken up by ASCT2 is consecutively exchanged to stimulate leucine uptake which in turn supports mTOR activation. SLC7A5 (also known as LAT1) ensures the efflux of L-glutamine and the leucine inward flux [13]. Most glutamine transporters including ASCT2 have been reported to be glycosylated but whether inhibition of glucose uptake and glycolysis influences the capacity of leukemia cells to utilize glutamine through alterations of the glycosylating process, is unknown.

In this study, we have examined how glucose withdrawal and direct inhibition of N-glycosylation by tunicamycin could influence ASCT2 glycosylation. This work led us to document how these treatments profoundly altered ASCT2 glycosylation but also to identify the plasticity of glutamine transport in the studied leukemia cells. We also identified LAT1 upregulation as a leukemia cell response to the inhibition of glycosylation and validated that the blockade of LAT1 could accentuate the growth inhibitory effects resulting from glycosylation inhibition.

## RESULTS

### Glucose and glutamine are required to maintain leukemia cell growth

**Figure 1:**
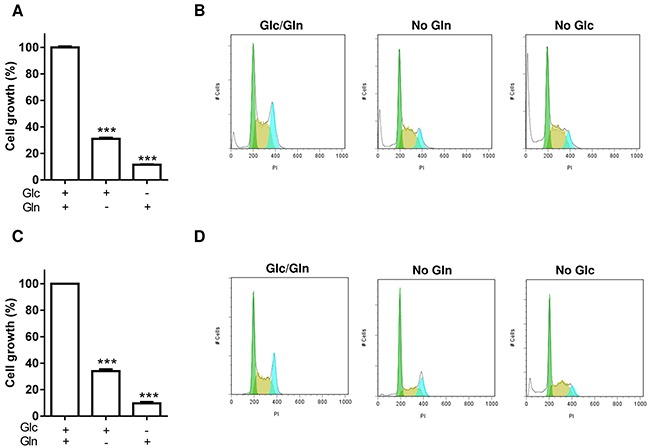
Glc and Gln are both necessary to maintain leukemia cell growth Leukemia cells were incubated for 48 hours in media containing either Glc or Gln or both. **A.** HL-60 and **C.** K-562 cell growth was measured using PrestoBlue (***P<0.001, n=3). Representative cell cycle study for **B.** HL-60 and **D.** K-562 cells revealing the accumulation of cells in subG1 phase and the reduction in G2/M phase when either Glc or Gln was lacking; this experiment was repeated three times with similar results.

In a first set of experiments, we documented the importance of glucose (Glc) and glutamine (Gln) to maintain the growth of leukemia cells of distinct origins, namely promyelocytic HL-60 and erythromyeloblastoid K-562 cells. Leukemia cell growth was significantly reduced after 48 hours of culture in the absence of Glc or Gln compared to the control condition both in HL-60 (Figure 1A) and K-562 cells (Figure 1C). Analysis of cell cycle confirmed that both Gln and Glc deprivation led to a reduction in G2/M phase coincident with a net increase in the subG1 phase (21.5% ± 4.0 and 29.0% ± 4.3, respectively *vs.* 4.0% ± 0.8 in control conditions for HL-60 cells and 9.5% ± 2.1 and 23.5% ± 5.5, respectively *vs.* 2.2% ± 0.8 in control conditions for K-562 cells) (Figure 1B and 1D).

### Glucose availability regulates glycosylation of the glutamine transporter ASCT2

**Figure 2:**
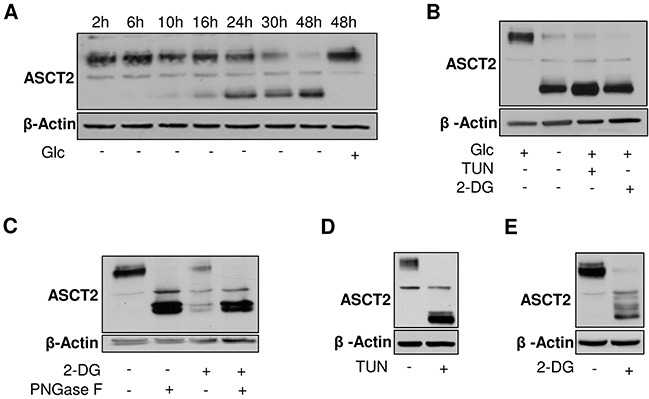
Glycosylation of ASCT2 is dependent on glucose availability Representative immunoblots from HL-60 leukemia cell extracts depicting **A.** the time course of ASCT2 deglycosylation in response to glucose withdrawal, **B.** the extent of ASCT2 deglycosylation in the absence of glucose and upon treatment with tunicamycin (TUN) or 2-deoxyglucose (2-DG) and **C.** the similar shift to lower MW obtained in the presence of 2-DG or PNGase F. Representative immunoblots from K-562 leukemia cell extracts depicting the extent of ASCT2 deglycosylation upon **D.** TUN or **E.** 2-DG treatment. These experiments were repeated 2-3 times with similar results; β-actin immunobloting was used as loading control.

To evaluate whether a defect in glycosylation could influence Gln metabolism, we first examined whether ASCT2 (SLC1A5), described as the main Gln transporter in cancer cells [20], could be affected by Glc deprivation. We found that in the absence of Glc, HL-60 cells exhibited over time a shift of ASCT2 immunoblot signal towards lower molecular weight (MW) (Figure 2A). We then cultured leukemia cells in the presence of two glycosylation inhibitors, namely tunicamycin (TUN) and 2-deoxyglucose (2-DG). TUN blocks GlcNAc phosphotransferase (GPT), which catalyzes the transfer of N-acetylglucosamine-1-phosphate from UDP-GlcNAc in the first step of glycoprotein synthesis while 2-DG blocks glycosylation by depleting cell in available glucose and via incorporation in various dolichol oligosaccharides that cannot be elongated anymore. Interestingly, the lower ASCT2 MW band observed upon glucose deprivation was also observed when HL-60 cells were exposed to either inhibitor (Figure 2B). We also treated HL-60 cell extracts with Peptide-N-Glycosydase F (PNGaseF), an enzyme able to cleave the bond between oligosaccharides and asparagine residue of the N-linked glycoprotein [21]. PNGaseF treatment led to the expected band shift, confirming that Glc deprivation promoted ASCT2 deglycosylation (Figure 2C). Similar results were obtained with K-562 leukemia cells exposed either to TUN (Figure 2D) or 2-DG (Figure 2E).

### Glycosylation and expression of ASCT2 are mostly dispensable for glutamine uptake in HL-60 and K-562 leukemia cells

**Figure 3:**
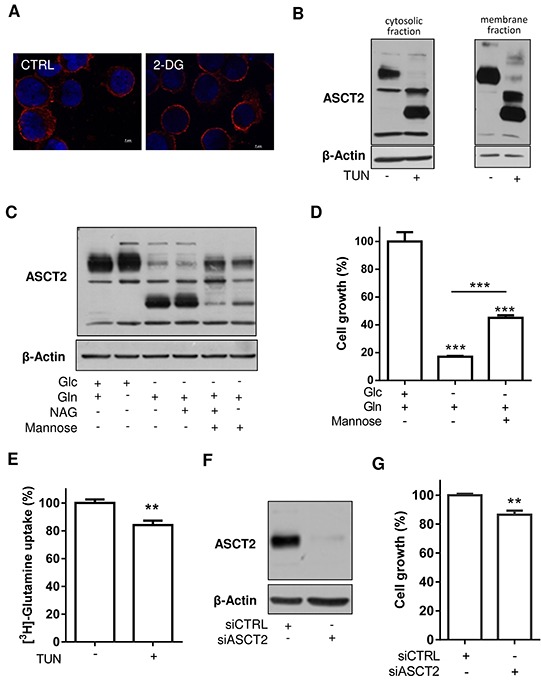
Inhibition of ASCT2 glycosylation and expression limitedly influences HL-60 leukemia cell growth **A.** ASCT2 immunofluorescence (red) detection in HL-60 cells treated or not with 2-deoxyglucose (2-DG); nuclei were counterstained with DAPI (blue). **B.** ASCT2 immunoblots from membrane-enriched and cytosolic compartments of tunicamycin-treated HL-60 cells (corresponding to bound and unbound streptavidin-biotin fractions, respectively). **C.** ASCT2 immunoblots depicting the effects of N-acetylglucosamine (NAG) and/or mannose to restore the glycosylation process in glucose-deprived HL-60 cells. **D.** Effects of exogenous mannose on glucose-deprived HL-60 cell growth (**P<0.01). **E.** Effects of tunicamycin on radiolabeled glutamine uptake in HL-60 cells (**P<0.01, n=3). **F.** Representative immunoblot depicting ASCT2 silencing upon exposure to dedicated siRNA. **G.** Inhibition of HL-60 cell growth after 48 hours post-treatment with ASCT2 siRNA (**P<0.01, n=3). Immunoblots were repeated 2-3 times with similar results; β-actin immunobloting was used as loading control.

To investigate the potential impact of ASCT2 deglycosylation on the cell phenotype, we first checked the subcellular location of the transporter by immunofluorescence. 2-DG treatment failed to show a change in ASCT2 subcellular location in HL-60 leukemia cells (Figure 3A). The unaltered plasma membrane location of deglycosylated ASCT2 was confirmed in a biotin-based fractionation assay where deglycosylated ASCT2 (upon tunicamycin treatment) was identified both in the cytosolic and membrane fractions of HL-60 cells (Figure 3B). We then exposed HL-60 cells to N-acetylglucosamine (NAG) and mannose in order to bypass the need of glucose to support the cellular process of glycosylation. Mannose in particular partly restores ASCT2 glycosylation (Figure 3C) and cell growth was also less inhibited when this precursor of glycosylation was present (Figure 3D). Since the use of exogenous NAG/mannose can contribute to the restoration of glycosylation of many potential actors of cell metabolism, we further examined in a functional assay whether inhibition of glycosylation by tunicamycin was associated with a reduction in glutamine transport. Surprisingly, we found that tunicamycin treatment barely influenced the uptake of radiolabeled glutamine (Figure 3E), suggesting that other glutamine transporters could compensate for a possible deficiency in ASCT2. Such hypothesis was further supported by the poorly impacted growth of leukemia cells when ASCT2 was silenced in HL-60 (see Figure 3F and 3G) and K-562 leukemia cells (Supplementary Figure S1A and S1B), a pattern contrasting with the net growth inhibitory effects resulting from complete Gln deprivation (compare *no Gln* condition in Figure 1A and ASCT2 siRNA in Figure 3G).

### Inhibition of glycosylation is associated with changes in the expression of various Gln transporters in leukemia cells

**Figure 4:**
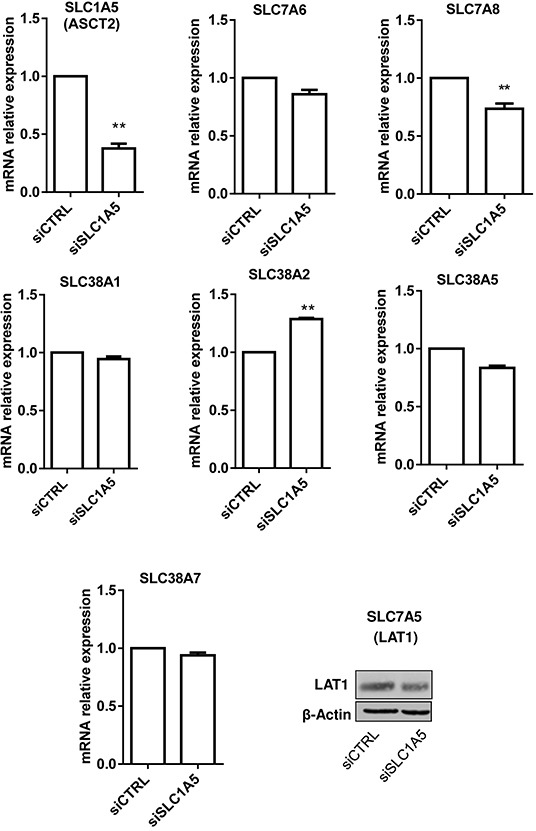
Silencing SLC1A5/ASCT2 marginally influences the expression of other glutamine transporters in HL-60 leukemia cells Effects of 48h HL-60 cell incubation with ASCT2/SLC1A5 siRNA on the mRNA (bar graphs) or protein expression (immunoblot) of the indicated glutamine transporters (**P<0.01, n=3).

As a first screen for possible compensatory Gln transport when ASCT2 is non-functional, we first checked whether ASCT2 silencing could lead to the upregulation of other Gln transporters known to be expressed in HL-60 cells (see Table 1) [13]; we used RT-PCR for detecting corresponding mRNA except for LAT1 for which a validated antibody was available. This strategy identified SLC38A2 as the only transporter *upregulated* in the absence of ASCT2 (Figure 4). Silencing SLC38A2 however had no impact on HL-60 cell growth even when ASCT2 was simultaneously knocked down (Supplementary Figure S2). We then extended this screening by silencing separately each of the putative Gln transporters reported to be expressed in HL-60 cells and by looking for mutual changes in their expression levels. This experiment failed to document any significant cross-regulation between Gln transporters (Supplementary Figure S3).

**Table 1:** Gln transporters

Gln transporters	Putative Glycosylation sites	Substrates	Glutamine flux	Expression in HL60
SLC1A5 (ASCT2)	Asn163, Asn212	Neutral amino acids	Influx/Efflux	yes
SLC6A14 (ATB^0.+^)	Asn155, Asn163, Asn174, Asn189, Asn197, Asn202, Asn230, Asn302	Neutral and cationic amino acids	Influx	no
SLC6A15 (B^0^AT2)	Asn187, Asn213, Asn383, Asn394	Neutral amino acids	Influx (at low level)	no
SLC6A19 (B^0^AT1)	Asn158, Asn182, Asn258, Asn354, Asn368	Neutral amino acids	Influx	no
SLC7A5 (LAT1)	Asn49, Asn230, Asn340	Neutral amino acids	Influx/efflux	yes
SCL7A8 (LAT2)	Not described	Neutral amino acids	Efflux	yes
SLC7A6 (y^+^LAT2)	Not described	Neutral and cationic amino acids	Influx	yes
SLC7A7 (y^+^LAT1)	Asn325	Neutral and cationic amino acids	Influx	Almost undetectable
SLC7A9 (b^0.+^AT)	Not described	Neutral and cationic amino acids	Efflux	no mRNA detected
SLC38A1 (SNAT1)	Asn251, Asn257	Neutral amino acids	Influx	yes
SLC38A2 (SNAT2)	Asn258, Asn274	Neutral amino acids	Influx	yes
SLC38A3 (SNAT3)	Asn74, Asn247, Asn248, Asn252, Asn323	Neutral amino acids	Influx/Efflux	no
SLC38A5 (SNAT5)	Asn226	Neutral amino acids	Influx/Efflux	yes
SLC38A7 (SNAT7)	Not described	Neutral, cationic and anionic amino acids	Influx	yes
SLC38A8 (SNAT8)	Not described	Neutral, cationic and anionic amino acids	Influx	not determined

Predicted glycosylation sites (Asn = Asparagine), nature of substrates, flux direction and expression in HL-60 leukemia cells are presented [13, 14, 27, 28].

**Figure 5:**
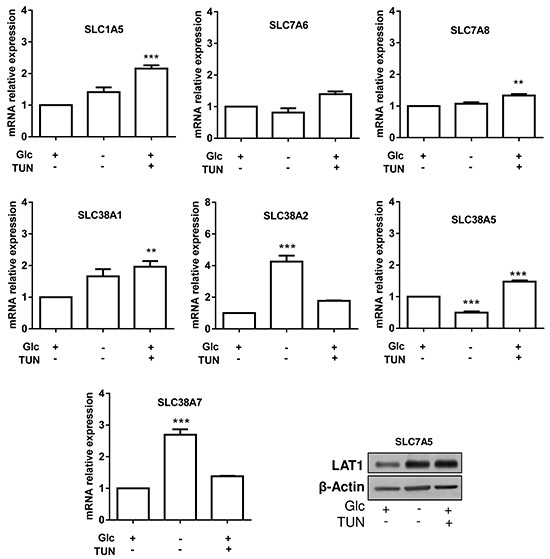
Glycosylation inhibition variably influences the expression of glutamine transporters in HL-60 leukemia cells Shown are the effects of 30h HL-60 cell incubation in the absence of glucose or in the presence of tunicamycin on the mRNA (bar graphs) or protein expression (immunoblot) of the indicated glutamine transporters (**P<0.01, ***P<0.001, n=3).

We then reasoned that changes in cell glycosylation pattern (instead of changes in expression) could directly influence the expression of Gln transporters in leukemia cells. We repeated the above screening in the absence of glucose and upon treatment with tunicamycin. We found that tunicamycin induced a significant increase in ASCT2 mRNA expression, supporting a possible compensatory mechanism for the altered glycosylation of ASCT2 (Figure 5). While the expression of the other transporters was upregulated in response to either tunicamycin treatment or glucose withdrawal (Figure 5), SLC7A5 (LAT1) was the only transporter exhibiting a net upregulation in both conditions (2.8 and 2.2-fold increase, respectively) (Figure 5).

### Upregulation of LAT1 attenuates the anti-proliferative effects resulting from glycosylation inhibition in leukemia cells

**Figure 6:**
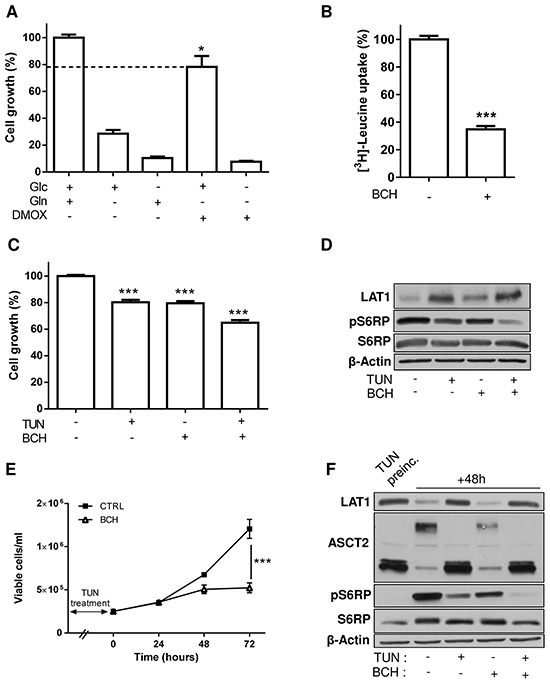
Inhibition of LAT1 increases the growth inhibitory effects resulting from alterations in the glycosylation process **A.** Cell-permeable dimethyloxoglutarate (DMOX) unmasks the contribution of non-metabolized glutamine to HL-60 cell growth. **B.** BCH blocks leucine uptake (**P<0.01, n=3). Additive inhibitory effects of 48h tunicamycin and BCH treatments on **C.** cell growth (***P<0.001, n=3) and **D.** S6RP phosphorylation. Effect of 72h cell prechallenge with tunicamycin on the effects of (consecutive) 48h BCH treatment **E.** on cell growth and **F.** S6RP phosphorylation. Immunoblots were repeated 2-3 times with similar results; β-actin immunobloting was used as loading control.

LAT1 is mostly described as part of a neutral amino acid exchanger involved in glutamine efflux in order to facilitate the uptake of leucine and the consecutive activation of the mTOR pathway [19, 22]. To study the contribution of glutamine to leucine uptake in our leukemia cell models, we first evaluated the effects of the cell membrane-permeable dimethyloxoglutarate (DMOX) as a source of alpha-ketoglutarate (αKG), a key intermediate of Gln early metabolism. We found a ∼20-30% reduction in HL-60 (Figure 6A) and K-562 cell growth (Supplementary Figure S1C) when glutamine was substituted by DMOX, confirming that non-metabolized glutamine plays a role in supporting leukemia cell growth (besides fueling TCA cycle with αKG). We then used 2-aminobicyclo-(2,2,1)-heptane-2-carboxylic acid (BCH), a well-known inhibitor of LAT1 that we validated using radiolabeled leucine uptake (Figure 6B); in these experiments, pharmacological inhibition was preferred to LAT1 silencing with siRNA to avoid the issue of the re-expression of the transporter with time. We observed that BCH reduced by ∼35% (p<0.01) the growth of HL-60 cells pretreated with tunicamycin (Figure 6C) (*vs.* 20% for each treatment administered separately). These effects were yet more pronounced when the extent of S6RP phosphorylation was measured to reflect the activation of the mTOR pathway. While BCH did not show significant effects on untreated HL-60 cells, BCH dramatically reduced the extent of phospho-S6RP in tunicamycin-exposed cells (−75.5% *vs.* untreated cells (P<0.01) and −30.5% *vs.* tunicamycin-treated cells (P<0.05)) (Figure 6D).

We then reasoned that the effects of LAT1 inhibition should be yet more striking in conditions wherein inhibition of glycosylation was just relieved, i.e. when upregulated LAT1 is avidly exploited for the inward flux of leucine and other neutral amino acids to support cell regrowth. We thus tested the effects of BCH on the growth of cells pretreated with tunicamycin. This experimental design confirmed that BCH prevented leukemia cell growth to a larger extent in cells pre-challenged with the glycosylation inhibitor (Figure 6E). These observations were validated by measuring the extent of S6RP phosphorylation in the different experimental conditions. BCH actually reduced the extent of phosphorylated S6RP by half in HL-60 pre-exposed for 72 hours to tunicamycin (compare lanes 2 and 4 in Figure 6F). When tunicamycin was maintained for the whole duration of the experiment, both cell growth (not shown) and S6RP phosphorylation (see lane 5 in Figure 6F) were fully abrogated. Inhibition of the mTOR pathway in response to tunicamycin was actually dependent on the time of exposure, amounting to ∼50% and ∼100% after 48 and 72 hours, respectively (see P-S6RP signal in lane 2 in Figure 6D and lane 1 in Figure 6F). Note also that tunicamycin withdrawal led to ASCT2 re-glycosylation after 48 hours (Figure 6F, lane 2).

## DISCUSSION

This study first provides the proof of concept that glucose deprivation by preventing the glycosylation of various proteins including those orchestrating cancer bioenergetics, may also alter glycolysis-independent metabolic routes in tumor cells. We showed indeed that inhibition of glucose metabolism in leukemia cells dramatically reduced the extent of glycosylation of ASCT2, a major transporter of glutamine, a key biosynthetic fuel in these cells [8, 9]. Although we failed to detect mislocalization of deglycosylated ASCT2, partial restoration of the cellular glycosylation pattern with mannose was associated with a significant increase in leukemia cell growth in the absence of glucose. Importantly, it was not only the complete glucose withdrawal but also the pharmacological inhibition of glycolysis by 2-deoxyglucose that led to a reduction in ASCT2 glycosylation. More generally, these data point towards a possible confusion in the interpretation of studies investigating the contribution of glucose (*vs.* other energy fuels) in cancer cell metabolism by either removing glucose from culture medium or using inhibitors of the upstream steps of glycolysis.

Interestingly, on our way to document a functional role of deglycosylated ASCT2 in leukemia cells HL-60 and K-562, we realized that complete ASCT2 silencing only marginally inhibited cell growth and glutamine uptake. This observation led us to consider the existence of a possible compensatory effect to counteract ASCT2 deglycosylation. Experiments aiming to probe a potential upregulation of glutamine transporters in response to ASCT2 silencing failed however to identify obvious candidates that could substitute for ASCT2; cross regulation between major Gln transporters was also not observed. Yet, when we looked for the effects of tunicamycin, a *bona fide* glycosylation inhibitor, on the expression of glutamine transporters, several of them showed a significant upregulation (namely ASCT2, SLC7A8, SLC38A1, SLC38A5, SLC7A5). Among the latter, only SLC7A5/LAT1 showed a significant overexpression in the presence of glucose withdrawal, strengthening a possible role of this transporter to counteract the inhibition of glycosylation in leukemia cells. This hypothesis is further supported by the lack of glycosylation modification of LAT1 [24], making it a good candidate to compensate for the functional deficiency of normally glycosylated transporters.

**Figure 7:**
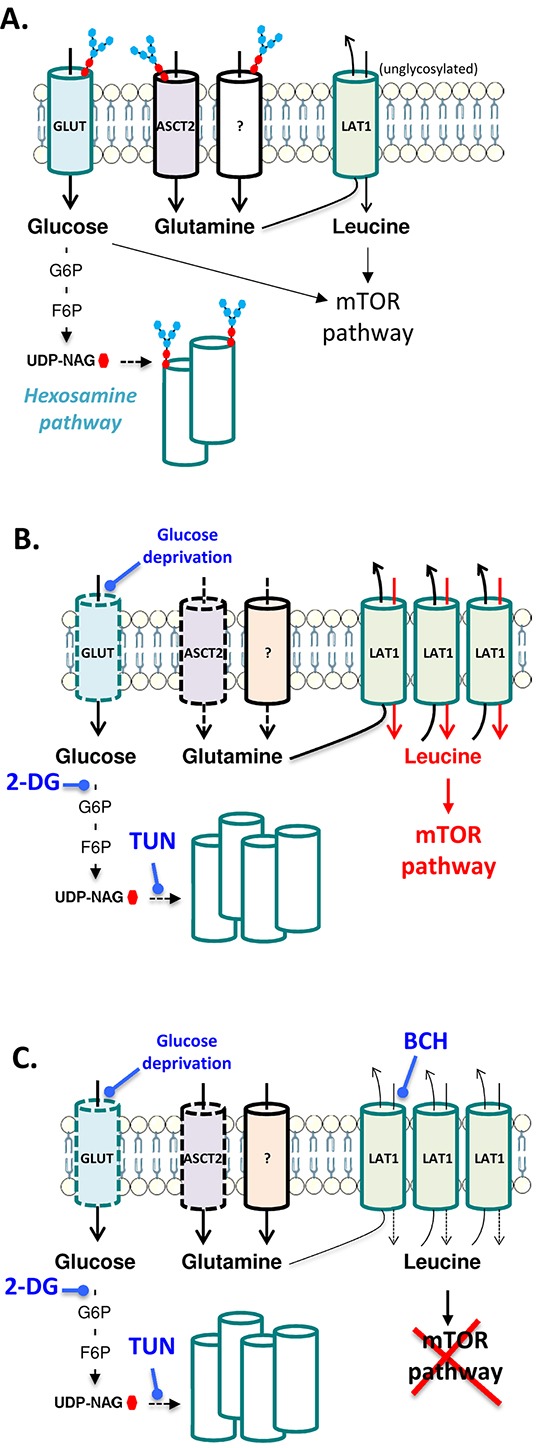
Schematic representation of the interplay between glucose metabolism and glutamine transporters in leukemia cells **A.** In normal growth conditions, glucose fuels the hexosamine pathway thereby contributing to the glycosylation of various nutrient transporters including glutamine transporters ASCT2 and others listed in Table 1. The mTOR pathway supporting cell growth is activated through the glucose metabolism and the influx of leucine facilitated by glutamine efflux via the LAT1 antiport. **B.** When glycosylation is inhibited in response to glucose deprivation or treatment with TUN or 2-DG, functional alteration of transporters is observed and leukemia cells compensate through the upregulation of various transporters including LAT1 that is a non-glycosylated transporter. Leukemia cells are then highly dependent on LAT1 to support the mTOR pathway. **C.** When the leucine uptake inhibitor BCH is combined to glycosylation inhibitors, mTOR is completely blocked and leukemia cell growth is dramatically inhibited.

LAT1 transports branched side-chain amino acids such as L-leucine into cells in exchange with L-glutamine [19, 23–25]. Nicklin and colleagues further proposed that cellular uptake of L-glutamine and its subsequent rapid efflux in the presence of essential amino acids is the rate limiting step that activates the mTOR pathway. Our observation of an upregulation of LAT1 in the presence of a slightly reduced glutamine uptake may thus represent a mechanism for leukemia cells to compensate for an alteration in glutamine homeostasis (see Figure 7). Adaptation to the inhibition of glycosylation of some Gln transporters including ASCT2 through recruitment of other Gln transporters may actually change the rate of glutamine flux thereby inducing LAT1 upregulation to counteract the lesser availability of intracellular glutamine. We cannot exclude however that transporters others than those involved in glutamine homeostasis could also be functionally altered in response to TUN treatment or that resulting ER stress indirectly contributed to LAT1 upregulation (even though such effects are more likely to occur upon prolonged exposure to the N-linked glycosylation inhibitor).

Although we could not document a change in subcellular location of ASCT2, we cannot exclude that a defect in ASCT2 glycosylation could induce subtle alterations in the extracellular domains of the transporter involved in glutamine recognition. It should also be emphasized that leukemia cells may behave differently than other cancer cells and that ASCT2 deglycosylation may lead to yet more pronounced effects in cancer cells where this transporter has a more exclusive role. For instance, loss of ASCT2 function in different solid tumor models either in response to pharmacological inhibition [19] or changes in subcellular location [26] was previously documented to lead to a powerful inhibition of cell growth. Differences may also exist between leukemia cells since ASCT2 blockade was reported to exert important antiproliferative effects in acute monocytic leukemia cells MOLM-14 [8]. Nevertheless, in the two leukemia cell lines evaluated in our study, we showed that inhibition of LAT1 further increased the growth inhibitory effects of tunicamycin, in particular post-treatment, i.e. when normal glycosylating conditions were restored. Combination of glycosylation inhibition and blockade of LAT1 showed a dramatic inhibition of the mTOR pathway (Figures 6D and 6F), underlying the therapeutic potential to combine glycolysis inhibitors with BCH or other leucine analogues (see Figure 7). Whether this strategy could be implemented in the clinics will depend on the potential toxic effects of this double-hit modality on healthy cells. Interestingly, we showed in experiments where we sequentially inhibited glycosylation and leucine uptake that profound cytostatic effects were still observed (see Figure 6E), indicating that drug regimen could be adapted to maintain treatment efficacy and reduce potential toxicities in non-tumor tissues.

In conclusion, the two major findings of this study are derived from the analysis of the consequences of glucose deprivation on the glycosylation of ASCT2. We first documented that leukemia cell bioenergetics may indirectly suffer from the inhibition of glucose metabolism through a profound effect on the glycosylation process. Although we mainly focused on ASCT2 for the proof of principle, this should be taken into account when dissecting the respective contribution of different nutrients for leukemia cell metabolism. Secondly, we identified that in the tested leukemia cells, ASCT2 is not the obligatory transporter of glutamine as reported in other types of cancer cells and LAT1 may partly rescue the effects of global cell deglycosylation. Altogether, these data point to the upregulation of LAT1 as a salvage pathway that should be targeted instead of the well known ASCT2 transporter, to complement the effects of glucose metabolism inhibitors in cancer treatment.

## MATERIALS AND METHODS

### Cell culture and reagents

Leukemia cell lines (HL-60, K-562) were cultured routinely in RPMI Glutamax (Life Technologies) supplemented with 10% FBS and 1% solution of 10,000 units/mL penicillin and 10,000 μg/mL streptomycin. Glucose or glutamine-deprived medium were obtained from DMEM powder (Sigma). In some experiments, cells were treated with 10 mM 2-deoxy-D-glucose (2-DG), 3 μg/mL Tunicamycin (TUN) and/or 10 mM 2-aminobicyclo-(2,2,1)-heptane-2-carboxylic acid (BCH); all these compounds were obtained from Sigma. L-[^3^H]-glutamine and L-[^3^H]-leucine were purchased from PerkinElmer.

### Cell density

Cells were seeded at 1×10^5^ or 2.5×10^5^ cells/ml. PrestoBlue^®^ (10%) was added to each well 2 hours prior fluorescence intensity measurement. Cells were also counted using Cellometer Auto T4 cell counter from Nexcelom. Viable cells were enumerated by Trypan blue exclusion.

### Cell cycle analysis

Cells were permeabilized using ethanol and propidium iodide was used to label DNA. BD FACScalibur was used to process the samples (1×10^4^ events/sample) and analysis was performed using FlowJo software 7.2.2.

### siRNA and transfection

The sequence of the siRNA targeting SLC1A5 (ASCT2) is CCGGUCCUGUACCGUCCUCAA, the one targeting SLC7A5 is UGCUAACGUCUUACUAAUUUA. They were obtained from Eurofins MWG Operon. The other siRNA sequences (SLC38A2, L-007559-01-0005); (SLC38A5, L-007562-02-0005); (SLC7A6, L-007616-01-0005) were purchased from Dharmacon. Leukemia cells were transfected using Amaxa Nucleofector kit V from Lonza according to the manufacturer's protocol; irrelevant siRNA was used as control.

### Western blotting

Immunoblotting experiments were carried out as previously described [9]. In some experiments, protein membrane isolation was first performed using EZ-link sulfo NHSS-biotin and streptavidin agarose resine (Thermo Scientific). Antibody directed against ASCT2 (Millipore) was used at a dilution of 1:2000. LAT1 antibody (1:1000) was purchased from Cell Signaling as well as antibodies against total S6RP (1:2000) and phosphorylated S6RP (Ser235/236) (1:2000).

### Immunofluorescence

Cells were fixed with PFA 4% for 10 minutes at room temperature. Cells were then re-suspended in water and were dropped off as a smear on a coverslip. Cells were permeabilized using Triton 0.1%; BSA 5% was used as blocking agent. The primary antibody ASCT2 was from Millipore (1:1000) and the secondary antibody was Alexa 568-conjugated (1:1000). DAPI was used to label the nucleus.

### Glutamine and leucine uptake

5×10^5^ cells were transferred from DMEM to Krebs solution in wells of masterblock plate (Greiner) and incubated with either L-[^3^H]-glutamine or L-[^3^H]-leucine. After aspiration on a 96-well filter plate and washing (3 times with Krebs solution), the radioactivity retained on the filters was counted in a microplate counter (PerkinElmer Topcount).

### RT-qPCR

RNA was extracted using the Maxwell RSC simply RNA Tissue kit (Promega). cDNA was synthesized from 2 μg of RNA using Revertaid reverse transcriptase with oligo-dT and random hexamers according to manufacturer's instructions (Thermo Scientific). The qPCR was performed using FAST-cycling parameters on a Viia7 (Life Technologies) using SYBRgreen CFX reagent (Life Technologies). Forward and reverse primers for qPCR were obtained from Eurogentec with the following sequences: *SLC38A2*, 5′-TCCTGTTAAGTGGTGTACTGGT-3′ 5′-CCAGGTG CATTGTGTACCCA-3′; *SLC38A5*, 5′-CAGGCATCCGA GCCTATGAG-3′ 5′-CCCCAACATTGTGCAGACAG-3′; *SLC1A5*, 5′-GAGCTGCTTATCCGCTTCTTC-3′, 5′-GGG GCGTACCACATGATCC-3′; *SLC6A19*, 5′-ACAGGGT ATGTGGACGAGTG-3′; 5′-GTGGAGATGTTGAGCG TCTCT-3′; *SLC6A14*, 5′-ACCGTGGTAACTGGT CCAAAA-3′, 5′-CGCCTCCACCATTGCTGTAG-3′; *SLC6A15*, 5′-GGAACTCTCTGTGGGTCAAAG-3′, 5′-CCGCCCAGTTTAGGGCTTA-3′; *SLC7A8*, 5′-AGGC TGGAACTTTCTGAATTACG-3′, 5′-ACATAAGCGAC ATTGGCAAAGA-3′; *SLC38A1*, 5′-AACCTCCTTAG GCATGTCTGT-3′, 5′-GCAAAGGCGAGTCCCAA AAT-3′; *SLC38A3*, 5′-GGAGCTGTATGGAGGG CAAG-3′, 5′-GAACACTGACATCCCGAATGAT-3′; *SLC7A6*, 5′-ATCGGGATTGCCCTTTCTGG-3′, 5′-CTG GTGATAGCAGCCAGGAC-3′′; *SLC38A7*, 5′-AAGAG CAGGTTGAGAAGAGTCC-3′; 5′-GAGCCTTCTTCC TGCAAGGT-3′.

### Statistical analysis

Data are represented as mean ± SEM of at least three independent experiments unless otherwise indicated. Student *t*-test or one-way ANOVA (Bonferroni's *post hoc* test) tests were used for statistical analysis. *P <0.05, **P <0.01, ***P <0.001

## SUPPLEMENTARY FIGURES


