# Supplementary material for: Inhibition of glucose metabolism prevents glycosylation of the glutamine transporter ASCT2 and promotes compensatory LAT1 upregulation in leukemia cells
Source: Oncotarget. 2016 Jun 17;7(29):46371–83. doi: 10.18632/oncotarget.10131 (PMC5216804; doi:10.18632/oncotarget.10131)
Supplement: Supplementary file 1 [file oncotarget-07-46371-s001.pdf]

Inhibition of glucose metabolism prevents glycosylation of the glutamine transporter ASCT2 and promotes compensatory LAT1 upregulation in leukemia cells

SUPPLEMENTARY FIGURES

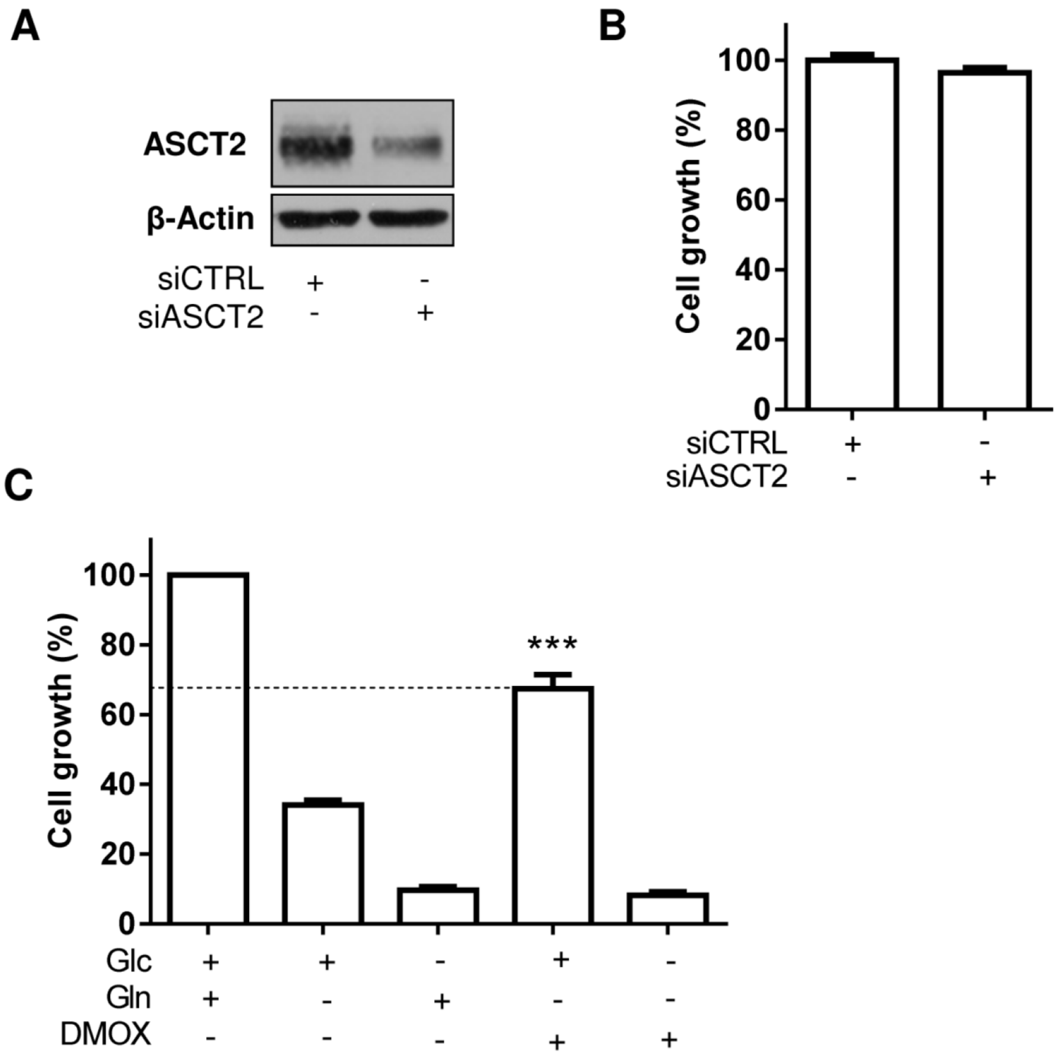

Supplementary Figure S1: ASCT2 silencing does not alter K-562 cell growth and DMOX does not completely compensate for glutamine deprivation. A. Representative immunoblot depicting ASCT2 silencing upon K-562 cell exposure to dedicated siRNA. Immunoblot was repeated two times with similar results; β-actin immunobloting was used as loading control. **B.** Inhibition of K-562 cell growth after 48 hours post-treatment with ASCT2 siRNA (**P<0.01, n=3). **C.** Cell-permeable dimethyloxoglutarate (DMOX) unmasks the contribution of non-metabolized glutamine to K-562 cell growth.

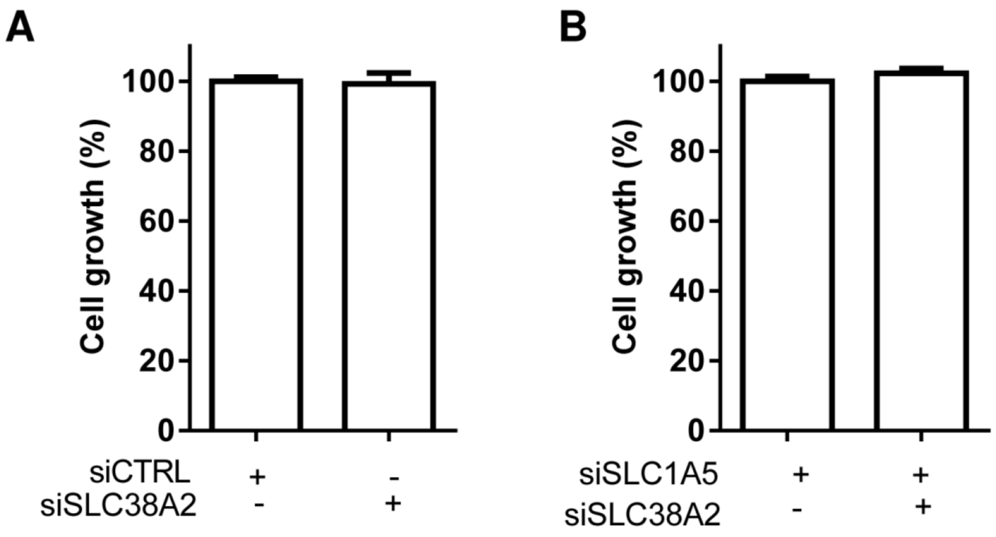

Supplementary Figure S2: SLC38A2 silencing does not inhibit HL-60 cell growth. Bar graphs show the effects of 48h incubation with SLC38A2 siRNA **A.** alone or **B.** in combination with SLC1A5/ASCT2 siRNA on HL-60 cell growth. These experiments were repeated once with similar results.

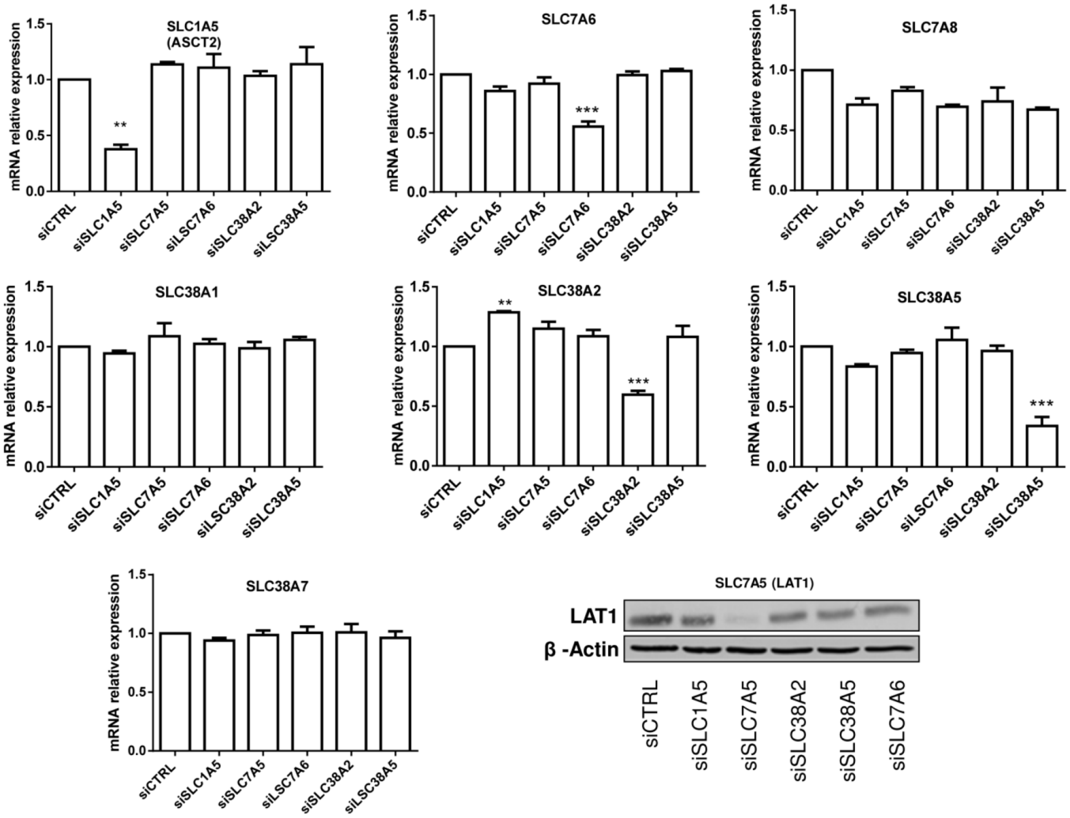

Supplementary Figure S3: Silencing various Gln transporters expressed in HL-60 cells does not influence their mutual expression. Shown are the effects of 48h HL-60 cell incubation with the indicated siRNA on the mRNA (bar graphs) or protein expression (immunoblot) of the indicated glutamine transporters (**P<0.01, ***P<0.001, n=3).
